# Clinical Progress in Inoperable or Recurrent Advanced Gastric Cancer Treatment from 1004 Single Institute Experiences Between 2007 and 2018

**DOI:** 10.1093/oncolo/oyab069

**Published:** 2022-02-19

**Authors:** Izuma Nakayama, Daisuke Takahari, Keitaro Shimozaki, Keisho Chin, Takeru Wakatsuki, Mariko Ogura, Akira Ooki, Daisaku Kamiimabeppu, Hiroki Osumi, Eiji Shinozaki, Kensei Yamaguchi

**Affiliations:** Department of Gastroenterological Chemotherapy, Cancer Institute Hospital of the Japanese Foundation for Cancer Research, Tokyo, Japan; Department of Gastroenterological Chemotherapy, Cancer Institute Hospital of the Japanese Foundation for Cancer Research, Tokyo, Japan; Department of Gastroenterological Chemotherapy, Cancer Institute Hospital of the Japanese Foundation for Cancer Research, Tokyo, Japan; Department of Gastroenterological Chemotherapy, Cancer Institute Hospital of the Japanese Foundation for Cancer Research, Tokyo, Japan; Department of Gastroenterological Chemotherapy, Cancer Institute Hospital of the Japanese Foundation for Cancer Research, Tokyo, Japan; Department of Gastroenterological Chemotherapy, Cancer Institute Hospital of the Japanese Foundation for Cancer Research, Tokyo, Japan; Department of Gastroenterological Chemotherapy, Cancer Institute Hospital of the Japanese Foundation for Cancer Research, Tokyo, Japan; Department of Gastroenterological Chemotherapy, Cancer Institute Hospital of the Japanese Foundation for Cancer Research, Tokyo, Japan; Department of Gastroenterological Chemotherapy, Cancer Institute Hospital of the Japanese Foundation for Cancer Research, Tokyo, Japan; Department of Gastroenterological Chemotherapy, Cancer Institute Hospital of the Japanese Foundation for Cancer Research, Tokyo, Japan; Department of Gastroenterological Chemotherapy, Cancer Institute Hospital of the Japanese Foundation for Cancer Research, Tokyo, Japan

**Keywords:** advanced gastric cancer, clinical trial, clinical practice, chemotherapy, platinum doublet

## Abstract

**Background:**

In the past decade, several successful clinical trials provided new therapeutic agents approved for advanced gastric cancer (AGC). This study evaluated whether these practice-changing results actually altered the clinical practice.

**Patients and Methods:**

We retrospectively reviewed medical records of treatment-naive AGC patients who received combination chemotherapy of fluoropyrimidine and platinum between 2007 and 2018 and divided them into three groups: Groups A (2007-10), B (2011-14), and C (2015-2018), respectively. We compared the clinicopathological features, treatment details, and clinical outcomes among the three groups.

**Results:**

In total, 1004 consecutive patients were enrolled (A; *n* = 254, B; *n* = 300, and C; *n* = 450). The number of patients with poor performance status, older age, esophagogastric junction adenocarcinoma, and primary tumor increased during the study period. All groups had similar median overall survival (OS); ~16 months) without any statistical difference but steady prolongation of survival was observed in the adjusted with imbalance prognostic factors among groups (B/A; hazard ratio, HR 0.82, 95% C.I 0.68-0.98, C/A; HR 0.72, 95% CI 0.60-0.86); OS of HER2-positive AGC patients was clearly improved (HER2-positive vs HER2-negative in Group B, HR 0.80, 95% CI 0.60-1.06; Group C, HR 0.68, 95% CI 0.51-0.90) but that of diffuse-type AGC patients remained dismal.

**Conclusions:**

The increasing availability of chemotherapy options potentially contributed to improved survival of AGC patients, but expanded chemotherapeutic indications made the survival benefit inconspicuous in the whole population. Future therapeutic development for the AGC subset not adequately receiving benefit from previous clinical trials is warranted.

## Teaser

Recent successful clinical trials resulted in approval of new therapeutic agents for advanced gastric cancer. This article evaluates whether these practice-changing results actually altered clinical practice.

**Implications for Practice**

The highlight of clinical practice over the past decade demonstrated that practice-changing clinical trials of AGC could actually modify clinical practice. Successful clinical trials provided patients with more opportunities to receive chemotherapy and helped some patients avoid radical noncurative gastrectomy and achieve long-term survival. However, progress in clinical practice did not simply translate into prolonged survival in the whole population. A clear advance in HER2-positive AGC refined the right target therapy could prolong a patient survival. Conversely, survival of patients with diffuse-type AGC remained dismal and a novel therapeutic development for diffuse-type AGC is urgently warranted.

## Introduction

Gastric carcinoma is estimated to be the fifth most common malignancy and the third leading cause of cancer-related mortality worldwide.^1^ Inoperable or recurrent advanced gastric cancer (AGC) confers a dismal prognosis; systemic chemotherapy is the standard of care for this patient population; this treatment improves their survival and quality of life.^2^

Several successful clinical trial advances provided increasing chemotherapeutic options for AGC patients since 2007. To date, there is no international common standard first-line treatment for AGC patients but a global consensus for fluoropyrimidine and platinum combination chemotherapy was reached in 2010.^3-8^ In Japan, based on the results of the Japan Clinical Oncology Group 9912 and SPIRITS trials, S1 plus cisplatin (SP) has been used as the first-line standard regimen since 2007.^7,8^ Concurrently, in Europe, the REAL-2 trial demonstrated that epirubicin, capecitabine, and oxaliplatin (EOX) combination had similar efficacy as the combination of epirubicin, infused fluorouracil, and cisplatin (ECF), which had been widely used in that region.^9^ In 2010, the ToGA trial demonstrated the superior efficacy of adjunctive trastuzumab to platinum-doublet chemotherapy in human epidermal growth factor receptor-2 (HER2)-positive gastric carcinoma.^10^ The establishment of platinum-doublet therapy as a first-line AGC treatment generated further clinical interest in subsequent therapy. Several clinical trials demonstrated prolonged survival compared with the best supportive care (BSC), and second-line chemotherapy was recognized globally as the standard of care between 2011 and 2014.^11-13^ The positive result of the RAINBOW trial established paclitaxel plus ramucirumab as the globally preferred regimen in AGC refractory to platinum-doublet therapy.^14^ Moreover, immune oncology (IO) for AGC patients has been included as salvage-line treatment since 2017. ATTRACTION-2 rial showed nivolumab monotherapy to have a statistically significant improvement in overall survival (OS) compared with placebo.^15^ Nivolumab is the third- or later-line treatment option for AGC in Japan, regardless of the expression of programmed death ligand 1 (PD-L1). In the USA, Pembrolizumab was approved based on the results of KEYNOTE-059 and -158 for selected AGC patients with positive biomarkers, such as high PD-L1 expression (≥1%) in the combined positive score (CPS), microsatellite instability-high (MSI-high), or tumor mutation burden-high (TMB-high).^16^ The success of the TAGS trials added trifluridine/tipiracil (FTD/TPI) to the salvage-treatment options.^17^ The DESTINY Gastric-01 trial demonstrated trastuzumab deruxtecan (T-DXd) as an active agent for third- or later-line therapy for HER2-positive AGC in 2020,^18^ and irinotecan could be an active candidate in this setting.^19^

Currently, Japanese AGC patients could receive systemic chemotherapy at most in the fifth or sixth (if HER2-positive) line. Thus, clinicians are greatly concerned to ensure that these practice-changing results actually improve their routine clinical practice. This study was conducted to review the clinical practices in the past 12 years and to elucidate how successful clinical trials have influenced routine clinical practice. The insight from this large-scale clinical experience is expected to provide a basis for the future development of clinical trials in the treatment of AGC patients

## Materials and Methods

### Patients Grouping and Treatment

**Figure 1.**
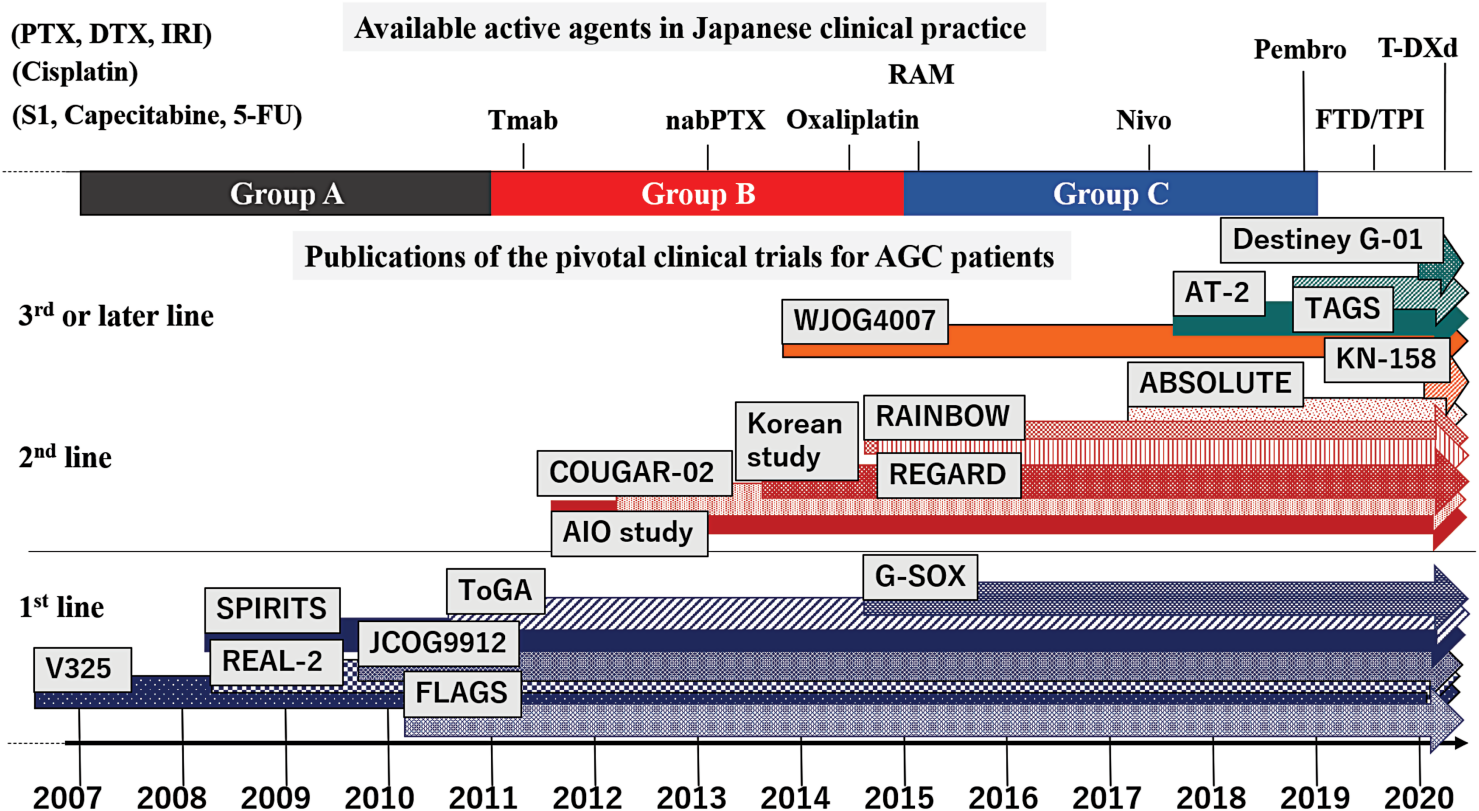
The overview of approved agents in Japan and accumulating evidences of pivotal trials for the advanced gastric cancer patients between 2007 and 2020. The year when each agent was available in Japanese clinical practice is indicated on the scale; S1, capecitabine, 5-FU, PTX and DTX (before 2007), Tmab (March, 2011), nab-PTX (February, 2013), oxaliplatin (September, 2014), RAM (June, 2015), Nivo (September, 2017), FTD/TPI (August, 2019), Pembro (December, 2019), and T-DXd (September, 2020). The colored arrows indicate the evidences which were basis for approval of agents above. The arrows begin at the year when each article of the pivotal trial was published. FU, fluorouracil; PTX, paclitaxel; DTX, docetaxel; Tmab, trastuzumab; nab-PTX, nanoparticle albumin-bound (nab)-paclitaxel; RAM, ramucirumab; NIVO, nivolumab; FTD/TPI, trifluridine/tipiracil; Pembro, Pembrolizumab; T-DXd, Trastuzumab deruxtecan; JCOG, Japan Clinical Oncology Group; AIO, Arbeitsgemeinschaft Internistische Onkologie; AT-2, ATTRACTION-2; KN-158, KEYNOTE-158.

This retrospective study was conducted at the Cancer Institute Hospital of the Japanese Foundation for Cancer Research (JFCR), Japan. We reviewed clinical data between January 2007 and December 2018 and enrolled patients who met the following criteria: (1) had histologically proven inoperable advanced or recurrent esophagogastric junction (EGJ) and stomach adenocarcinoma, (2) received combination chemotherapy containing fluoropyrimidine and platinum, (3) received no prior systemic chemotherapy for metastasis, and (4) provided written informed consent for receiving the treatment. We excluded patients with other types of advanced tumors and those with early recurrence (<6 months after the final administration of neoadjuvant or adjuvant chemotherapy). To explore the impact of pivotal clinical trials on clinical practice, we assessed the data from 2007 when platinum-doublet chemotherapy was established as the standard of care in Japan. The calendar period was segmented into three groups (Groups A, B, and C) of 4-year intervals between 2007 and 2018 for analysis. Approved agents and published years of pivotal trials are summarized in Figure 1. The treatment schedule and dosage were followed as specified in pivotal clinical trials. This study was approved by the ethics committee of Cancer Institute Hospital of JFCR (approval no. 2019-1082) and was conducted in accordance with the tenets of the Declaration of Helsinki of 1964 and later versions. Given the retrospective nature of this study, informed consent for observational study was waived with the opportunity to opt out from participation.

### Statistical Analysis

We defined OS as the time from the start of chemotherapy to death or the latest follow-up. Progression-free survival (PFS) was defined as the time from the start of chemotherapy to death or the first day of disease progression, as determined by imaging or clinical examination. The cut-off date was March 17, 2021. We used Kaplan–Meier survival curves to calculate the OS, PFS, and PPS and used the log-rank test for intergroup comparison of the clinical outcomes. The proportion of the subsequent chemotherapy (PSC) was calculated by dividing the number of patients who received subsequent therapy by the total number of patients who received subsequent chemotherapy and BSC. Proportion of the subsequent chemotherapy-X was the proportion of patients who received X-line chemotherapy after (X − 1)-line therapy. We compared categorical data using two-sided Fisher’s exact tests. Multivariate analysis was conducted using a Cox regression model. Covariates with a *P*-value <.05 in the univariate analysis were chosen for the multivariate analysis. The stratified Cox proportional hazards regression model was used to calculate the adjusted hazard ratio (HR) with independent prognostic factors, which were significantly imbalanced among the three groups. For all analyses, *P* < .05 was considered statistically significant. All statistical analyses were performed using a graphical user interface for R (The R Foundation for Statistical Computing, Vienna, Austria), GraphPad Prism ver. 9.0 for Windows (GraphPad Software, San Diego, CA), and the JMP version 14.2.0 software (SAS Institute, Cary, NC).

## Results

### Patient Characteristics

Between January 2007 and December 2018, 1132 consecutive patients with inoperable advanced or recurrent gastric carcinoma were administered combination therapy of fluoropyrimidines and platinum at the Cancer Institute Hospital of the JFCR, Tokyo, Japan. Among them, 41 patients had early recurrence within 6 months after neoadjuvant or adjuvant chemotherapy, and 32 patients had other types of advanced tumors. Fifty-five patients started systemic chemotherapy at other hospitals. Ultimately, 1004 consecutive patients were enrolled in this study, and 254, 300, and 450 patients were classified into Group A, B, and C, respectively (Supplementary Figure S1). The total number of AGC patients treated with platinum-containing regimens showed a gradually increasing trend during the study period. The baseline patient characteristics are listed in Table 1. The proportion of elderly patients (age ≥75 years) and those with EGJ carcinoma gradually increased from Group A (2.0% and 14.2%), B (4.7% and 18.7%), to C (9.3% and 22.7%), respectively. The proportion of patients with recurrent AGC increased along with the study periods (A: 11.8%, B: 16.0%, and C: 20.0%), although the proportion of patients who underwent gastrectomy before systemic chemotherapy decreased (A: 37.0%, B: 36.0%, and C: 26.2%). Among the study groups, Group C had the highest proportion of patients with peritoneal metastases (C: 52.0%; A: 36.6%, and B: 37.0%); however, the proportion of diffuse-type AGC was consistent among the three groups (A: 60.9%, B: 64.1%, and C 65.6%). Group C had the highest proportion of patients with poor Eastern Cooperative Oncology Group performance status (ECOG PS: PS≥1, 42.7%; A: 32.6% and B: 37.0%). S1 was mainly chosen as the first-line fluoropyrimidine regimen throughout the study periods (70.4-93.3%). S1 plus cisplatin was predominantly administered in the initial period (*n* = 230, 90.6%); although after 2015, oxaliplatin-containing regimens replaced cisplatin in our clinical practice (Group A, 2.4% vs Group B, 9.7% vs Group C, 82.9%). The proportions of HER2-positive AGC were similar in groups B (25.3%) and C (20.7%).

**Table 1.** Baseline characteristics of the patients (*n* = 1004).

	Group A 2007-2010 (*n* = 254)	Group B 2011-2014 (*n* = 300)	Group C 2015-2018 (*n* = 450)	*P*-value
** **Age				
Median (range)	61 (16-78)	62 (30-82)	66 (21-84)	
Age 75 years	5 (2.0)	14 (4.7)	42 (9.3)	**<.001**
Sex, *n* (%)				
Male	179 (70.5)	185 (61.7)	292 (64.9)	**.089**
Female	75 (29.5)	115 (38.3)	158 (35.1)	
ECOG, *n* (%)				
PS 0	218 (87.9)	200 (67.1)	258 (57.3)	**<.001**
PS ≥1	30 (12.1)	98 (32.9)	192 (42.7)	
Unknown	6 (2.4)	2 (0.7)	0 (0.0)	
Lauren classification, *n* (%)				
Intestinal	100 (39.4)	107 (35.9)	155 (34.4)	**.401**
Diffuse	153 (60.2)	190 (63.3)	295 (65.6)	
Unknown	1 (0.4)	3 (1.0)	0 (0.0)	
Location of primary site, *n* (%)				
EGJ/cardia	36 (14.2)	56 (18.7)	102 (22.7)	**.020**
Stomach	216 (85.0)	234 (78.0)	342 (76.0)	
Unknown	2 (0.7)	10 (3.3)	6 (1.3)	
*HER2* status, *n* (%)				
Negative	47 (18.5)	217 (72.3)	351 (78.0)	**<.019**
Positive	10 (3.9)	76 (25.3)	93 (20.7)	
Unknown/not assessed	197 (77.6)	7 (2.3)	6 (1.3)	
Extent of disease *n* (%)				
Metastatic	216 (85.0)	243 (81.0)	353 (78.4)	**<.020**
Locally advanced	8 (3.1)	9 (3.0)	7 (1.6)	
Recurrent	30 (11.8)	48 (16.0)	90 (20.0)	
Prior gastrectomy, *n* (%)				
Yes	94 (37.0)	108 (36.0)	118 (26.2)	**.002**
No	160 (63.0)	192 (64.0)	332 (73.8)	
Metastatic site, *n* (%)				
Liver	79 (31.1)	81 (27.0)	115 (25.6)	.28
Distant LN	97 (38.2)	81 (27.0)	164 (36.4)	**.007**
Peritoneum	93 (36.6)	111 (37.0)	234 (52.0)	**<.001**
Ovary	7 (2.8)	9 (3.0)	14 (3.1)	.965
Bone	14 (5.5)	15 (5.0)	37 (8.2)	.16
Lung	20 (7.9)	19 (6.3)	19 (4.2)	.121
No. of mestastases, *n* (%)				
2>	175 (68.9)	233 (77.7)	291 (64.6)	**<.001**
≥2	79 (31.1)	67 (22.3)	159 (35.3)	
Fluoropyrimidine, *n* (%)				
S1-based regimen	236 (92.9)	217 (72.3)	317 (70.4)	**<.001**
Capecitabine-based regimen	18 (7.1)	80 (26.7)	101 (22.4)	
5-FU-based regimen	0 (0.0)	3 (1.0)	32 (7.1)	
Platinum, *n* (%)				
CDDP-based regimen	248 (97.6)	261 (87.0)	62 (13.8)	**<.001**
L-OHP-based regimen	6 (2.4)	29 (9.7)	373 (82.9)	
Trastzumab, *n* (%)	0 (0.0)	81 (27.0)	87 (19.3)	

Abbreviations: EGJ, esophagogastric junction; ECOG, Eastern Cooperative Oncology Group; PS, performance status; LN, lymph node; HER2, human epidermal growth factor type 2; SP, S-1 plus cisplatin; SOX, S-1 plus oxaliplatin; XPT, capecitabine, cisplatin plus trastuzumab; FPT, fluorouracil, cisplatin plus trastuzumab.

Bold values indicate if a *P*-value was statistically significant (≤.05).

### Treatment Details During the Study Periods

**Figure 2.**
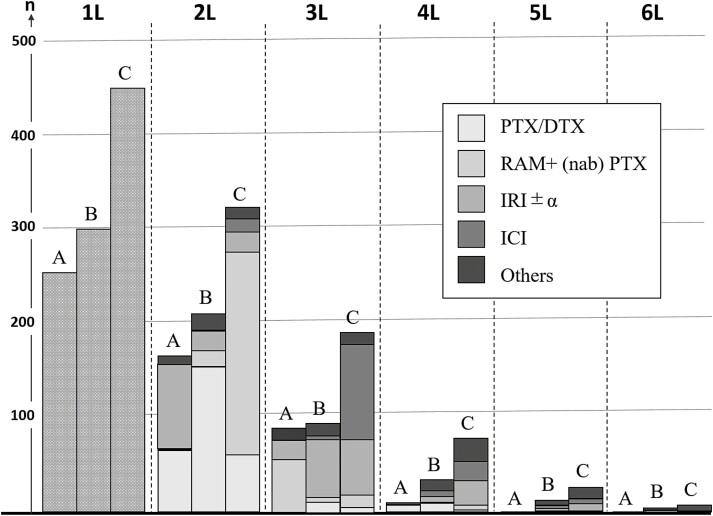
The number of patients treated in each line of chemotherapy by study groups. This bar graph shows the patient number (vertical axis) at each line of chemotherapy (horizontal axis) by study period. A total number of patients were increasing by age at any line of chemotherapy. Only limited number of patients received third or later line of chemotherapy especially in groups A and B. The preferentially chosen treatment regimens were different among study period in second- and third-line treatment.

As per the cut-off date, the total number of patients in each line of chemotherapy is shown in Figure 2, and the details of the regimens are summarized in Table 2. By the cut-off date, 7, 10, and 31 patients in Groups A, B, and C, respectively, had ongoing first-line treatment or were transferred to other hospitals on treatment. Thus, 247 (97.2%), 290 (96.7%), and 419 (93.1%) patients in Groups A, B, and C, respectively, had confirmed discontinuation of their first-line chemotherapy. The PSC-2 gradually increased from Groups A (73.0%), B (77.2%) to C (81.9%). The preferred second-line regimen changed during the study period. Between 2011 and 2014, paclitaxel or docetaxel replaced irinotecan as the second-line chemotherapy; since 2015, ramucirumab plus (nab)-paclitaxel was the most selected second-line chemotherapy. Proportion of the subsequent chemotherapy-3 was highest in Group C (66.4%) and lowest in Group B (47.5%). In Group B, 62% of patients received irinotecan-based therapy as third-line treatment; however, instead of irinotecan (30.7%), immune checkpoint inhibitors (52.6%) were administered preferentially as third-line treatment in Group C. The number of patients who received fourth-line or later-line chemotherapy was very small in Groups B and C. Interestingly, various surgical approach changes were apparent. The proportion of metastasectomy was similar among the three groups, although the proportions of patients who underwent gastrectomy before or after chemotherapy decreased during the study period (Group A: *n* = 123, 48.4%; Group B: *n* = 125, 41.7%; and Group C: *n* = 144, 32.0%). Furthermore, among patients who underwent gastrectomy, non-curative or palliative surgery was less likely to be performed after 2015 (Group A: *n* = 81, 31.9%; Group B: *n* = 70, 23.3%; Group C: *n* = 32, 7.1%). Conversion surgery rates were similar among the study groups (A: *n* = 13, 5.9%; B: *n* = 10, 3.3%; and C: *n* = 24, 5.3%).

**Table 2.** Treatment details according to each line of therapy.

Goup A (2007-10)														
line	total	PTX/DTX	RAM+(nab-)PTX	IRI based	ICI	Other regimens	CTx total	PSC-2	PSC-3	PSC-3	PSC-4	PSC-5	BSC	others
1L	254	—	—	—	—	—	—	—	—	—	—	—	—	7
2L	247	65 (39.4)	1 (0.6)	90 (54.5)	0 (0.0)	9 (5.4)	165	73.0%	—	—	—	—	61	21
3L	165	58 (63.7)	0 (0.0)	20 (22.0)	0 (0.0)	13 (14.3)	91	—	56.5%	—	—	—	70	4
4L	91	7 (70.0)	0 (0.0)	1 (10.0)	0 (0.0)	2 (20.0)	10	—	—	11.6%	—	—	76	5
5L	10	1 (100.0)	0 (0.0)	0 (0.0)	0 (0.0)	0 (0.0)	1	—	—	—	11.1%	—	8	1
6L	1	0 (0.0)	0 (0.0)	0 (0.0)	0 (0.0)	0 (0.0)	0	—	—	—	—	0.0%	1	0
Goup B (2011-2014)														
line	total	PTX/DTX	RAM+(nab-)PTX	IRI based	ICI	Other regimens	CTx total	PSC-2	PSC-3	PSC-3	PSC-4	PSC-5	BSC	others
1L	300	—	—	—	—	—	—	—	—	—	—	—	—	10
2L	290	154 (73.3)	17 (8.1)	21 (10.0)	1 (0.5)	17 (8.1)	210	77.2%	—	—	—	—	62	28
3L	210	11 (11.6)	5 (5.3)	62 (65.3)	4 (4.2)	13 (13.7)	95	—	47.5%	—	—	—	105	10
4L	95	3 (10.7)	1 (3.6)	6 (21.4)	6 (21.4)	12 (42.9)	28	—	—	30.1%	—	—	65	2
5L	28	0 (0.0)	1 (8.3)	3 (25.0)	3 (25.0)	5 (41.7)	12	—	—	—	42.9%	—	16	0
6L	12	0 (0.0)	0 (0.0)	0 (0.0)	2 (50.0)	2 (50.0)	4	—	—	—	—	33.3%	8	0
Goup C (2015-2018)														
line	total	PTX/DTX	RAM+(nab-)PTX	IRI based	ICI	Other regimens	CTx total	PSC-2	PSC-3	PSC-3	PSC-4	PSC-5	BSC	others
1L	450	—	—	—	—	—	—	—	—	—	—	—	—	31
2L	419	61 (19.2)	215 (67.8)	10 (3.2)	19 (6.0)	12 (3.8)	317	81,9%	—	—	—	—	70	63
3L	317	6 (3.1)	13 (6.8)	59 (30.7)	101 (52.6)	13 (6.8)	192	—	66.4%	—	—	—	97	28
4L	192	2 (2.6)	5 (6.4)	26 (3.3)	20 (25.6)	25 (32.1)	78	—	—	42.6%	—	—	105	9
5L	78	1 (3.8)	0 (0.0)	8 (30.7)	5 (19.2)	12 (46.2)	26	—	—	—	37.7%	—	43	9
6L	26	0 (0.0)	0 (0.0)	0 (0.0)	1 (14.3)	6 (85.7)	7	—	—	—	—	31.8%	15	4

Abbreviations: PTX, paclitaxel; DTX, docetaxel; IRI, irinotecan; ICI, immune checkpoint inhibitor; CTx, chemotherapy; PSC, proportion of the subsequent.

### Clinical Outcomes: OS and PFS

**Figure 3.**
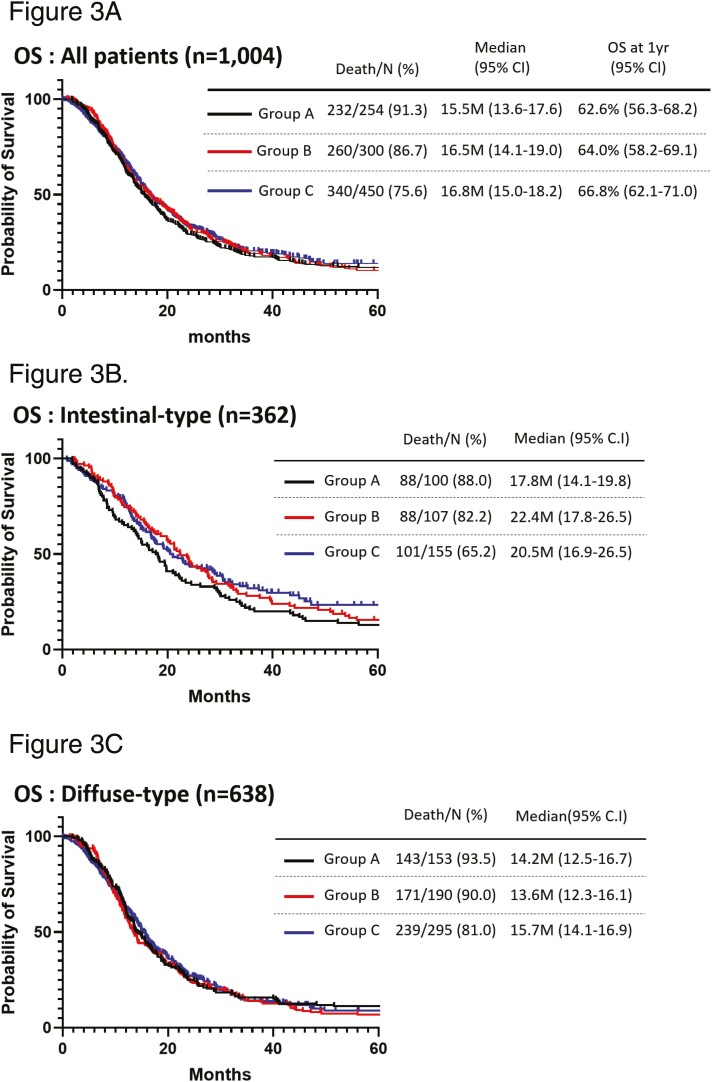
(**A**) Kaplan-Meier curves of OS according to the study periods (black line; 2007-2010, red line; 2011-2014, blue line; 2015-2018) in the whole population (*n* = 1004). There was no statistically significant difference in OS of whole population between any two groups. (B) Kaplan-Meier curves of OS according to the study periods (black line; 2007-2010, red line; 2011-2014, blue line; 2015-2018) in the intestinal-type patients (*n* = 362). (**C**) Kaplan-Meier curves of OS according to the study periods (black line; 2007-2010, red line; 2011-2014, blue line; 2015-2018) in the diffuse-type patients (*n* = 637). Trend toward statistically significant difference in OS of intestinal-type AGC was observed between groups A and B or C. However, Kaplan-Meier curves of OS of diffuse-type AGC were almost identical.

**Figure 4.**
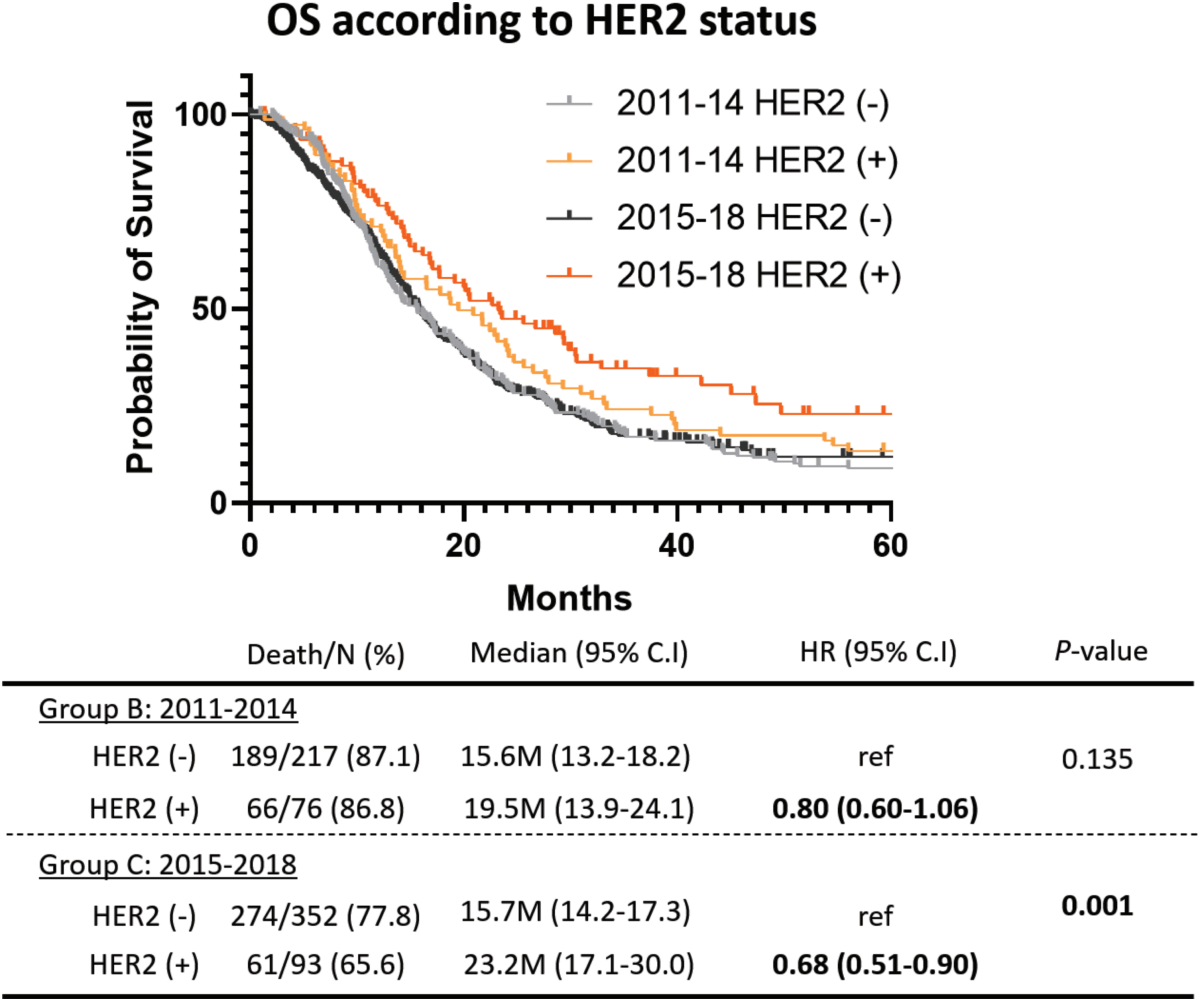
Comparison of Kaplan-Meier curves of OS between HER2-positive and HER2-negative advanced gastric cancer (light grey line: HER2-negative in 2011-2014, light orange line: HER2-positive in 2011-2014, grey line: HER2-negative in 2015--2018, orange line: HER2-positive in 2015-2018). The HR for mortality in HER2-positive AGC, compared with HER2-negative AGC, improved between groups B and C.

All treatment follow-ups were completed by March 17, 2021. The median follow-up time of the patients on the cut-off date was 15.3, 15.8, and 15.1 months (Groups A, B, and C, respectively). By the date of the analysis, 232 (91.3%), 260 (86.7%), and 340 (75.6%) patients in Groups A, B, and C, respectively, had died, and the median (95% confidence interval [95% CI]) of OS was 15.5 (13.6-17.6), 16.5 (14.1-16.0), and 16.8 (15.0-18.2) months, respectively. No statistically significant differences in OS were observed between any two groups (Figure 3A). At the cut-off date, 220 (86.6%), 256 (83.6%), and 392 (87.1%) patients in Groups A, B, and C, respectively, had disease progression; the median (95% CI) PFS was similar for the three groups (7.5 [6.5-8.5], 7.5 [6.9-8.7], and 7.3 [6.5-8.4] months, respectively; Supplementary Figure S2). A statistically significant prolongation of both OS and PFS was not observed among the three groups. However, subgroup analyses revealed the prolongation of survival during the study period. The OS of intestinal-type AGC in Groups B and C showed trends toward improvement when compared with that of Group A (B vs A: HR: 0.82, 95% CI: 0.61-1.11, *P* = .200/ C vs A: HR: 0.77, 95% CI: 0.57-1.02, *P* = .064) (Figure 3B). Conversely, Kaplan-Meier curves of the OS of diffuse-type AGC were almost identical and, there were no differences between the three groups (Figure 3C). A clear improvement in the initial treatment for HER2-positive AGC was observed in 2011. There was no significant difference in the median OS between Groups B and C in the HER2-negative AGC population (B: 15.6 months [95% CI: 13.2-18.2] vs C: 15.7 months [95% CI: 14.2-17.3 months]; Figure 4). However, the HR for mortality in HER2-positive AGC, compared with HER2-negative AGC, improved between Groups B and C (Group B: HR: 0.80 [95% CI: 0.60-1.06] vs Group C: HR 0.68 [95% CI: 0.51-0.90]; Figure 4). Numerical differences of median OS between HER2-positive and HER2-negative AGC extended from 3.9 months in Group B to 7.5 months in Group C. The median OS of HER2-positive AGC patients reached 23.2 months (95% CI: 17.1-30.0) in Group C. Since the approval of oxaliplatin for clinical use in Japan in 2017, a total of 30 patients in Group C received FOLFOX, and the median OS was only 5.4 months (95% CI: 3.8-10.8). No patient received FOLFOX as the first-line setting in Groups A and B.

### Univariate and Multivariate Analysis

The results of univariate and multivariate analyses are summarized in Table 3. Multivariate analysis demonstrated that ECOG PS (≥1), diffuse-type histology, no prior gastrectomy, 5-FU based regimen, elevated serum ALP levels (>ULN), higher NLR levels (≥4.0), and peritoneum metastasis had independent prognostic values for shorter survival (Table 3). Among the three groups, there were statistically significant disparities in the proportion of patients with poorer PS (≥1), 5-FU-based regimen, prior gastrectomy, higher NLR, and peritoneum metastasis. Thus, we calculated adjusted HR for survival with above mentioned five factors as co-variants to compare the OS between each of the two groups. Consequently, there were statistically significant differences in OS between Groups A and B (adjusted HR 0.82, 95% CI 0.68-0.98, *P* = .033) and A and C (adjusted HR 0.72, 95% CI 0.60-0.86, *P* < .001). The OS tended to be improved in Groups B and C (adjusted HR 0.88, 95% CI 0.74-1.03, *P* = .115).

**Table 3.** Univariate and multivariate analysis.

Variable	Univariate analysis			Multivariate analysis		
	HR	95% CI	*P*-value	HR	95% CI	*P*-value
Age (<75) vs 75≤	1.26	(0.94-1.68)	.127			
Sex male vs female	1.05	(0.91-1.21)	.527			
ECOG PS 0 vs 1≤	1.61	(1.39-1.86)	**<.001**	1.38	(1.18-1.62)	**<.001**
Primary site; stomach vs EGJ	0.83	(0.70-0.99)	**.044**	0.90	(0.75-1.09)	.300
Lauren classification; intestinal vs diffuse	1.43	(1.23-1.65)	**<.001**	1.42	(1.21-1.68)	**<.001**
Prior gastrectomy; no vs yes	0.71	(0.62-0.83)	**<.001**	0.80	(0.68-0.94)	**.006**
Disease status; metastatic vs recurrence	0.88	(0.73-1.06)	.168			
Fluoropymidine; S1 or Cape vs 5-FU	3.15	(2.21-4.50)	**<.001**	1.95	(1.33-2.84)	**<.001**
Platinum; oxaliplatin vs cisplatin	1.08	(0.93-1.24)	.314			
Serum ALP level;≤ULN vs ULN<	1.63	(1.38-1.93)	**<.001**	1.36	1.12-1.64	**<.002**
NLR; 4< vs 4≤	1.66	(1.43-1.92)	**<.001**	1.33	1.13-1.57	**<.001**
Metastatic site						
Liver mets; no vs yes	1.14	(0.98-1.33)	.085			
Peritoneum mets; no vs yes	1.38	(1.20-1.58)	**<.001**	1.19	(1.02-1.39)	**.031**
Bone mets; no vs yes	1.54	(1.18-2.01)	**.001**	1.02	(0.75-1.37)	.913
Lung mets; no vs yes	1.11	(0.83-1.46)	.485			
Ovary mets; no vs yes	0.85	(0.55-1.30)	.447			
distant LN mets; no vs yes	1.06	(0.92-1.22)	.447			
No. of metastatic site; ≤1 vs 2≤	1.37	(1.18-1.59)	**<.001**	1.18	(0.99-1.40)	.061

Abbreviations: ECOG, Eastern Cooperative Oncology Group; PS, performance status; EGJ, esophagogastro junction; ALP, alkaline phosphatase; NLR, neutrophil-to-lymphocyte ratio; ULN, upper limit of normal; No, number.

Bold values indicate if a *P*-value was statistically significant (≤.05).

### Treatment Details of Long-Term Survivors

We defined a long-term survivor as the patient that was alive for more than 3 years from the start of the initial systemic chemotherapy. At the cut-off date, 157 patients met the definition. The proportions of long-term survivors did not differ much among the three groups (A: *n* = 45, 17.7%; B: *n* = 52, 17.3%; and C: *n* = 60, 13.3%). However, the treatment, which long-term survivors received gradually changed across the study period. Metastasectomy was performed in 10 (22.2%), 8 (15.4%), and 14 (23.3%) patients in Groups A, B, and C, respectively, whereas the proportions of patients who underwent gastrectomy decreased along the study period (A: 80.0%; B: 65.4%; and C: 63.3%). Furthermore, some patients had recurrent tumors after curative gastrectomy; 74 patients received no surgical treatment for metastatic or recurrent AGC. Only eight patients (17.7%) in Group A were included among those 74 patients, whereas 30 (57.7%) and 36 (60.0%) patients in Groups B and C, respectively, were among those 74 long-term survivors. Therefore, more long-term survivors received third-line or later-line chemotherapy in Group B (*n* = 12) and Group C (*n* = 10) than in Group A (*n* = 1). Thus, recently, long-term survival was achieved by the continuum of chemotherapy.

## Discussion

The highlight of 1004 clinical experiences for the past 12 years demonstrated that the increasing availability of chemotherapy options has expanded chemotherapy indications and altered the therapeutic strategy. Improvement of survival depended on the subsets of AGC patients. A clear advancement in prolonged survival was seen on HER2-positive AGC compared with HER2 negative, but no prolonged survival was observed among diffuse-type AGC patients throughout the study periods.

Drug lags between Western countries and Japan was an urgent issue in Japanese clinical oncology until the early 2010s, although this gap has closed at least in the field of gastrointestinal cancers.^5,20^ Furthermore, there are 15 available active agents and at most five (six, if HER2 positive) lines of recommended treatment regimens in the treatment options of AGC patient. Great successes in pivotal clinical trials provided new agents that changed the therapeutic strategy, including surgery in the past 12 years. Despite the emergence of evolving active agents for AGC patients, the median OS in all three groups was approximately 16 months, and statistically significant intergroup differences in OS and PFS were not observed. However, several studies using clinical trials and clinical practice data have previously demonstrated the importance of subsequent therapy to improve OS in AGC patients treated with first-line chemotherapy.^21-25^ Post progression-free survival (PPS) had more correlation with OS than PFS, and subsequent therapy administration would play a key role in prolonged survival of AGC patients.^22,24,26^ As shown in cases of metastatic colorectal cancer,^27^ some data supported the therapeutic strategy of administering active agents contributing to prolonged survival of AGC patients.^28^ Thus, most experts in this field would not doubt that the availability of more agents would provide longer survival. Clinicians manage to continue sequential chemotherapy after disease progression where possible.^29^ From this standpoint, our unexpected result would not be acceptable. Ascertaining why prolonged survival in the past 12 years was not observed in our clinical practice would be a great question for clinicians in this field. The possible reasons for the unexpected results could be as follows.

First, during the study period, there were changes in the indications for patients who could receive platinum-based therapy. Platinum-doublet therapy with cisplatin was mainly chosen in the initial two periods, whereas oxaliplatin rapidly replaced cisplatin after 2015.^30^ In general, a patient requires adequate renal and cardiovascular function to tolerate a cisplatin regimen. Furthermore, a patient with massive ascites or inadequate oral intake would be excluded as a recipient of the cisplatin regimen. However, due to less renal toxicity and the lack of necessity for hydration, oxaliplatin could be administered for some patients with the abovementioned unfavorable factors. In fact, a greater proportion of elderly patients (age ≥75 years) and those with poor ECOG PS were included in Group C than in the other two groups. All 30 patients with inadequate oral intake or massive ascites treated with FOLFOX were in Group C. The median OS of patients who received FOLFOX was much shorter than that of patients who received other platinum-based therapies. Second, the global establishment of second-line chemotherapy in early 2010 had little impact on our clinical practice. Before the several phase III trials conducted between 2011 and 2014,^11-13,25,31^ second-line therapy was already being administered as standard care in Japan.^32-35^ In fact, more than 70% of patients in Group A were treated with irinotecan- or taxane-based chemotherapy. According to the RAINBOW trial,^14^ ramucirumab plus paclitaxel became a new standard of care after 2015, and more patients received combination therapy with ramucirumab in Group C than in Group B. However, the addition of ramucirumab to paclitaxel could not demonstrate OS improvement in the Japanese subgroup of the RAINBOW trials.^36^ Therefore, an advantage of adjunctive ramucirumab was not observed in the OS from before to after 2015. Third, an increase in treatment options in the third-line or later-line setting would have limited efficacy for prolonging patient survival from that of first-line chemotherapy. Nivolumab demonstrated superior efficacy in OS to BSC but not to chemotherapy.^15^ Before 2015, patients eligible for third-line therapy were mainly administered taxane and irinotecan in groups A and B, respectively. Avelumab, an anti-PD-L1 antibody, failed to demonstrate superiority to the physician’s choice regimen in the Javelin Gastric-300 study.^37^ However, this study only showed no survival improvement of whole population in the past 12 years but, not deny the potential of increasing chemotherapy options for survival prolongation. Importantly, if the imbalance of baseline characteristics among groups was adjusted, the risk reduction for death was gradually observed during the study period. We believe the remarkable successes of clinical trials could provide survival benefit for the AGC patients in clinical practice. These are future issues to expand the survival benefit for more AGC patients and to achieve further improvement in survival time.

A remarkable advancement in the management of HER2-positive AGC was observed clearly in our clinical practice, which could be used for further improvement in this field. Deep tumor shrinkage and durable response enabled HER2-positive AGC patients to receive subsequent therapy after disease progression of first-line therapy with favorable conditions and a low tumor burden.^38^ Furthermore, according to the exploratory subgroup analysis of the ATTRACTION-2 study, patients previously treated with trastuzumab recorded a higher response rate (16.9%) than patients without trastuzumab (7.7%).^39^ A promising result with a combination of IO and anti-HER2 therapy was reported and several studies with IO therapy as front-line treatment for HER2-positive AGC are ongoing.^40^ Additionally, T-DXd was newly approved to treat HER2-positive AGC patients based on the results of DESTINY-GC 01 trial in Japan,^18^ which is promising for further improvement in the prognosis of HER2-positive AGC patients. Conversely, the prognosis of patients with diffuse-type AGC, which constitutes the majority of AGC in clinical practice, remains dismal. No numerical differences were found in OS of diffuse-type AGC among the three groups. The further differences in treatment outcomes were evaluated between those who received target therapy and those who did not. Further investigations of novel therapeutic targets, such as CLDN18.2 and CLDN18-ARHGAP fusion, are urgently warranted.^41-43^

“The more lines of chemotherapy, the better the outcome” would be true when second- or third-line regimens are being established. A recent systematic review and meta-analysis demonstrated a correlation between OS and the proportion of patients with subsequent chemotherapy after first- and second-line chemotherapy; all OSs of individual clinical trials were less than 15 months, which was the median OS achieved in Group A.^23^ Moreover, limited number of AGC patients receive a third- or later-line chemotherapy. Without reliable biomarkers, clinicians hardly utilize various therapies in salvage-line therapy of AGC. A large-scale population-based analysis (*n* = 6909) using cancer registry data of the Kanagawa prefecture supported a similar trend to that of our study. The 3-year survival rate of stage IV AGC patients with chemotherapy dramatically increased from 4.1% (1995-2000) to 10.2% (2007-2009) although an apparent slowdown of improvement was observed in 2007-2015.^44^ The results of these studies indicate that various therapies in the Japanese circumstances of salvage-line therapy for AGC patients might reach saturation. It would be inevitable that the majority of AGC patients would have symptomatic disorders after their second or third disease progression and many would not maintain a general condition that could tolerate systemic chemotherapy. We hope for the emergence of revolutionary monitoring or diagnostic methods, such as liquid biopsy, to detect disease progression at an appropriate time for further improvement of AGC treatment.

The change in attitude toward gastrectomy should be considered when evaluating the progress in clinical management during the study period. The incidence of noncurative or palliative surgery for stenosis or bleeding caused by a primary tumor decreased across the study period. Since 2015, the FOLFOX regimen could be administered as first-line treatment to patients with stenosis of the primary tumor. In second-line chemotherapy, the emergence of ramucirumab plus (nab)-paclitaxel with high response rates enabled control of local progression and contributed to the prevention of noncurative radical surgery.^14,45^ Furthermore, the negative result of the REGATTA study possibly made surgeons avoid primary tumor resection in AGC patients with a single metastasis.^46^ Increasing varieties of chemotherapy or treatment regimens with high response rates in the past decade could expand the chemotherapeutic options for the treatment of AGC. Systemic chemotherapy has increasingly become more significant.

This study has several limitations. First, it was conducted at a single Japanese institute. Therapeutic agents available in Japanese clinical practice are not approved in other countries. This study presents a model to elucidate the effect of several kinds of newly approved agents. Second, follow-up times of survivors were short to adequately evaluate the long-tail effect of nivolumab. Further investigation is required to evaluate the influence of IO therapy on the clinical management of AGC patients. Third, this study included no information about toxicity. Ideally, clinical progress should be evaluated with consideration on treatment-related adverse events but due to the retrospective nature, we could not adequately obtain these data. However, this study could highlight the clinical issue and offer insights into next-generation therapeutic development.

## Conclusion

The practice-changing results from several clinical trials actually changed our clinical practice. The increasing number of treatment options enabled the expansion of chemotherapy indications and changed the therapeutic strategy, including surgery, which caused disparities in patient characteristics among the study periods. Although the prolongation of survival over the past 12 years could not be observed in the whole population, a steady improvement of survival was achieved after adjusting disparities of prognostic factors among study groups. A remarkable success in HER2-positive AGC refined that advancement of the right targeted therapy could certainly translate into survival benefit of patients. Identifying a novel target for diffuse-type AGC and biomarkers for existing therapies, revolutionized diagnosis methods are desirable for developing our clinical practice to the next stage.

## Supplementary Material

oyab069_suppl_Supplementary_Figures

## Data Availability

The data underlying this article will be shared on reasonable request to the corresponding author.
