# Correction to: Clinical Progress in Inoperable or Recurrent Advanced Gastric Cancer Treatment from 1004 Single Institute Experiences Between 2007 and 2018

**DOI:** 10.1093/oncolo/oyac105

**Published:** 2022-05-21

This is a correction to: Nakayama I, Takahari D, Shimozaki K, et al. Clinical progress in inoperable or recurrent advanced gastric cancer treatment from 1004 single institute experiences between 2007 and 2018. *The Oncologist*. 2022 [Advance access publication 19 February 2022]. https://doi.org/10.1093/oncolo/oyab069

In the originally published version of this article, the multivariate analysis values in Table 3, lines “Serum ALP level; ≤ULN vs ULN<” and “NLR; 4< vs 4≤”, were incorrect. The errors were corrected for final publication.

