# Supplementary material for: Clinical Progress in Inoperable or Recurrent Advanced Gastric Cancer Treatment from 1004 Single Institute Experiences Between 2007 and 2018
Source: Oncologist. 2022 Feb 19;27(6):e506–17. doi: 10.1093/oncolo/oyab069 (PMC9177114; doi:10.1093/oncolo/oyab069)
Supplement: oyab069_suppl_Supplementary_Figures [file oyab069_suppl_supplementary_figures.pdf]

Supplemental Figures for:
Clinical progress in inoperable or recurrent advanced gastric cancer treatment from 1,004 single institute experiences between 2007 and 2018
Daisuke Takahari et al.

**Supplementary Figure S1 Patient selection flow diagram**

Abberations AGC; advanced gastric cancer, CTx; Chemotherapy, Pts; Patients

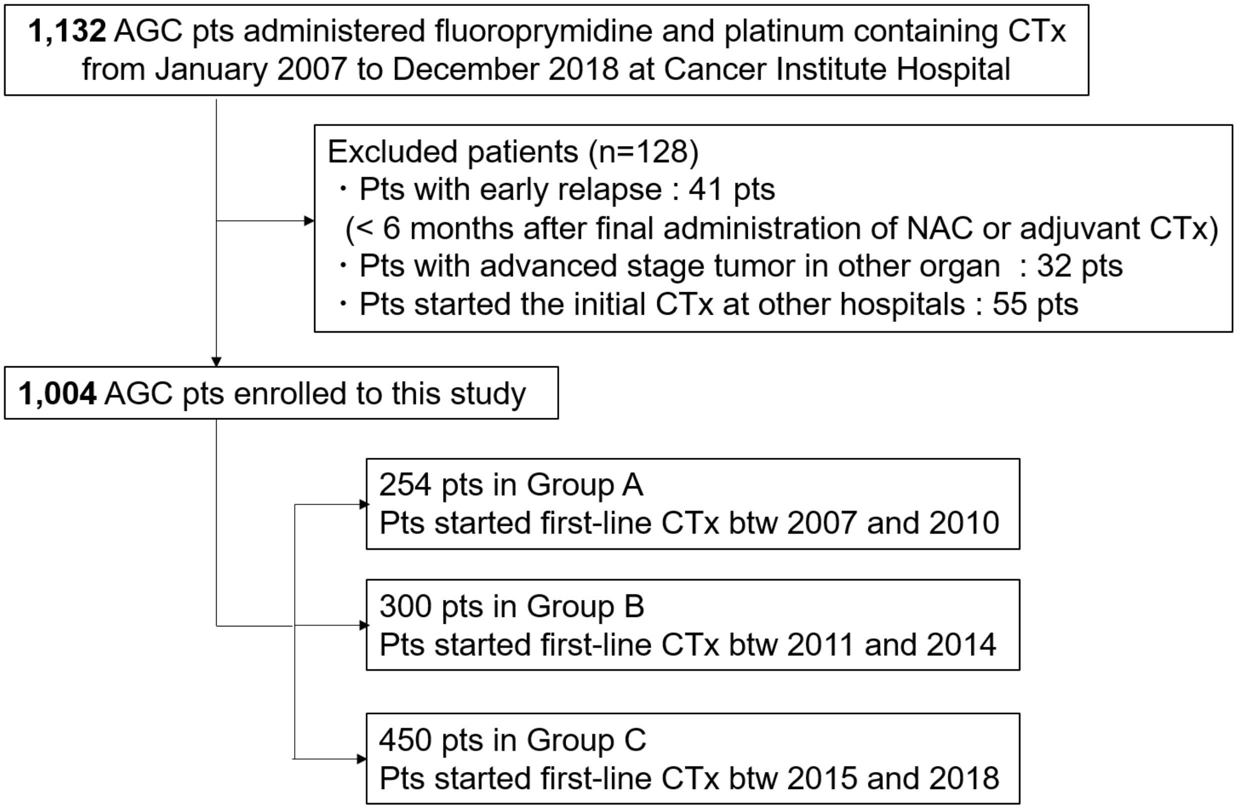

**Supplementary figure S2 Kaplan-Meier Curves of PFS according to the study periods (black line; 2007-2010, red line; 2011-2014, blue line; 2015-2018) in the whole population (n=1,004)**

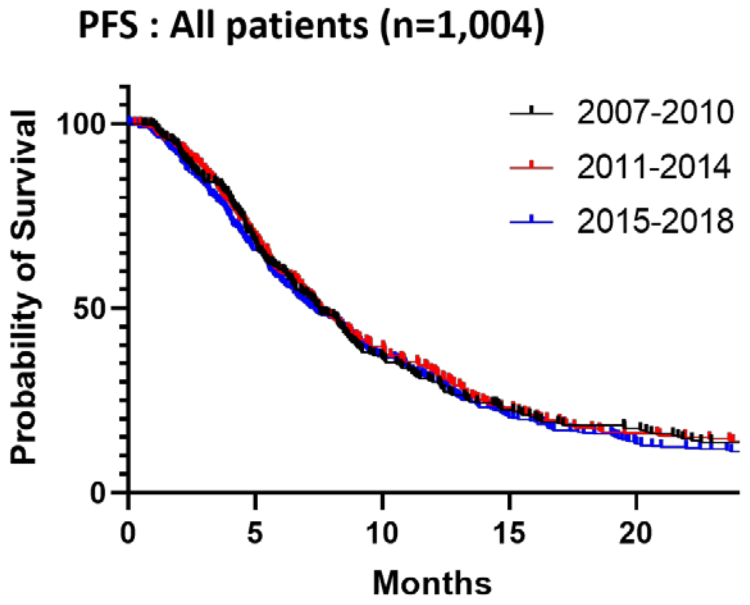


| | Death/N (%) | Median (95% C.I) |
|---|---|---|
| A: 2007-2010 | 220/254 (86.6) | 7.5M (6.5-8.5) |
| B: 2011-2014 | 256/306 (83.6) | 7.5M (6.9-8.7) |
| C: 2015-2018 | 391/450 (86.9) | 7.3M (6.5-8.4) |
